# Sepsis incidence and mortality in China, 1990–2021: An analysis for the Global Burden of Disease Study

**DOI:** 10.1016/j.jointm.2026.04.004

**Published:** 2026-05-06

**Authors:** Mingmin Pang, Qi Zhang, Haoyu Wang, Shihan Zhang, Yanan Li, Mengfei Lu, Miaosa Zhang, Hao Wang

**Affiliations:** 1 Department of Critical Care Medicine, Qilu Hospital of Shandong University, Jinan, Shandong, China; 2 Innovation Research Center for Sepsis and Multiple Organ Injury, Shandong University, Jinan, Shandong, China

**Keywords:** Sepsis, Global Burden of Disease, China

## Abstract

**Background:**

Sepsis, a life-threatening dysregulated host response to infection that causes organ dysfunction, remains a major source of preventable health loss worldwide. However, its burden in China has not been fully characterized. We therefore estimated sepsis incidence and mortality in China from 1990 to 2021 within the framework of the Global Burden of Disease (GBD) 2021 study.

**Methods:**

Using China-specific GBD 2021 anchor values together with publicly available GBD outputs, we reconstructed annual all-age sepsis incidence and mortality from 1990 to 2021 with piecewise log-linear interpolation between published anchor years. Broad cause-group burden was allocated using annual shares from the China GBD outputs. Because direct Chinese age-standardized anchor values were unavailable, age-standardized incidence and mortality were treated as sensitivity analyses. Piecewise linear and monotonic cubic Hermite (PCHIP) interpolation were also examined in sensitivity analyses.

**Results:**

In 2021, China had an estimated 6,773,717 incident sepsis cases (95% uncertainty interval [UI] 4,828,778–9,275,569) and 2,480,000 sepsis-related deaths (2,080,000–2,950,000). From 1990 to 2021, absolute numbers of incident cases increased, sepsis-related deaths declined modestly, and age-standardized incidence and mortality fell substantially. Infection-attributed sepsis remained the largest contributor to mortality, while the burden was disproportionately concentrated in males and older adults.

**Conclusions:**

Sepsis remains a major source of health loss in China. Despite declining age-standardized rates, the absolute burden remains large, with infection-attributed sepsis continuing to drive the greatest share of mortality and with older adults, males, and people with chronic diseases bearing disproportionate risk.

## Highlights

• Reconstructed China's sepsis burden using GBD 2021 and published China anchors.
• China had 6.77 million sepsis cases and 2.48 million deaths in 2021.
• Age-standardized incidence and mortality declined from 1990 to 2021.
• Absolute sepsis cases rose, while sepsis-related deaths declined modestly.
• Infection-attributed sepsis had the highest age-standardized burden in 2021.

## Introduction

Sepsis, defined as a life-threatening dysregulated host response to infection leading to organ dysfunction^[^^1^^]^ remains a major but incompletely quantified cause of preventable mortality and health loss.^[^^2^^,^^3^^]^ Although substantial progress has been made in sepsis recognition, standardized management, and infection prevention,^[^^4^^,^^5^^]^ the burden of sepsis in China is still difficult to characterize because sepsis is often recorded as an intermediate rather than an underlying cause of death and because standard national time-series outputs are not routinely available.

Recent Chinese studies and systematic syntheses have improved the evidence base,^[^^6^^,^^7^^]^ but each still captures only part of the national burden. A nationwide study of hospitalized sepsis showed that standardized hospital incidence rose from 328.25 per 100,000 in 2017 to 421.85 per 100,000 in 2019, with 57.5% of admissions occurring in adults older than 65 years,^[^^8^^]^ while a population-based mortality study showed persistent excess burden in males, older adults, and western China despite declining age-standardized mortality between 2006 and 2019. A recent study analyzing the sepsis burden in China using the Global Research on Antimicrobial Resistance (GRAM) framework further highlighted high mortality among older adults and infants and the growing importance of pathogen resistance.^[^^9^^]^ However, these studies do not provide the same transparent China-specific estimates of incidence and mortality within the published Global Burden of Disease (GBD) study 2021 framework, together with broad underlying-cause decomposition and harmonized national time trends.

At the same time, standard GBD outputs do not provide a directly interpretable China-specific sepsis time series, and the original individual-level datasets used in the GBD sepsis framework are not publicly accessible. A transparent China-focused reconstruction based on publicly available GBD outputs and published Chinese anchor values is therefore needed to clarify long-term national patterns without implying that the original GBD model was re-estimated. Accordingly, we used publicly available China-specific GBD 2021 results together with the Chinese anchors reported in the 2025 GBD sepsis paper to reconstruct sepsis incidence and mortality in China from 1990 to 2021.^[^^10^^]^ We aimed to describe temporal patterns in all-age burden, age-standardized incidence and mortality, variation by sex, age, and broad underlying cause category, and the robustness of the interpolated national series to alternative interpolation assumptions.

## Methods

### Study design and data sources

This study was a secondary analysis of sepsis burden in China from 1990 to 2021 based on publicly available GBD 2021 results and the material of the 2025 GBD sepsis paper.^[^^10^^]^ The working dataset consisted of China-specific GBD outputs by year, sex, age group, metric, and broad cause, together with Chinese all-age sepsis anchor values extracted from the published tables and a locally curated mapping of available China GBD causes into infections, non-communicable diseases, and injuries. We did not have access to the original individual-level multiple-cause-of-death or hospital datasets used by the GBD Collaborators, so all results should be interpreted as national estimates aligned to the published Chinese GBD sepsis results.

### Definition and analytical scope

Sepsis was defined according to the conceptual framework used in the 2025 GBD sepsis paper,^[^^10^^]^ in which sepsis is treated as a life-threatening dysregulated host response to infection that occurs as an intermediate event on the pathway to death or severe illness. In the present analysis, we did not reclassify explicit or implicit sepsis from raw ICD-coded records; instead, we adopted the published sepsis framework and estimated the corresponding China series from the reported national anchor values. This distinction is important because the present study is not a de novo re-estimation of the original GBD regression models.

The primary target outputs were incident sepsis cases, sepsis-related deaths, and their corresponding rates for China. For all-age counts and crude rates, we aligned the estimates to the China-specific values reported in the 2025 paper supplement. These tables provided the main reference values for 1990, 2019, and 2021.

### Calibration framework and key assumptions

Our estimates relied on three prespecified assumptions. First, annual incidence and mortality between published anchor years were interpolated with piecewise log-linear functions, implying proportional rather than absolute year-to-year change, exact preservation of the 1990, 2019, and 2021 anchor values, and no negative interpolated values. Second, grouped incidence and mortality were estimated by applying annual shares from the China GBD exports to the national totals aligned to the published anchors. Third, because the supplement did not report direct China age-standardized series, age-standardized incidence and mortality were presented as sensitivity analyses rather than as primary outputs.

### Broad underlying-cause groups

We used a three-group framework comprising infections, injuries, and non-communicable diseases. For grouped incidence, available China GBD causes in the local export were mapped to these three categories and annual group shares were normalized within year and then applied to the national incidence totals aligned to the published anchors. For grouped mortality, the infectious and non-infectious subtotals were taken directly from the 2025 paper supplement, and the non-infectious subtotal was apportioned between non-communicable diseases and injuries using shares from the China GBD aggregate outputs. We describe these results as group estimates based on China GBD shares rather than directly observed values.

### Sex-specific, age-specific, and age-standardized analyses

Sex-specific and age-specific estimates were derived by applying proportional distributions from the China GBD outputs to the national series aligned to the published anchors. For the 2021 age profile of sepsis-related deaths, age shares from the China GBD outputs were rescaled to the cause-specific mortality totals aligned to the published anchors. This approach preserved the age structure available in the China GBD outputs while maintaining consistency with the published national totals. We did not infer age-specific grouped incidence directly from the paper supplement.

### Age-standardized sensitivity analyses

Age-standardized incidence and mortality were handled separately because the published 2025 supplement provided all-age crude rates rather than direct China-specific age-standardized series. We therefore estimated age-standardized incidence and mortality in sensitivity analyses by retaining the long-term age-standardized trajectories from the China GBD outputs through 2019 and applying the relative 2019–2021 change observed in the crude-rate series aligned to the published anchors. To assess robustness, we repeated the national interpolation using piecewise linear and monotonic cubic Hermite interpolation and summarized differences against the main piecewise log-linear series (Supplementary Table S1).

### Statistical presentation

Results are presented as counts or rates with 95% uncertainty intervals (UI), following the published GBD sepsis reporting style.^[^^11^^]^ The main text emphasizes all-age incidence and mortality aligned to the published Chinese anchors, whereas the age-standardized analyses are clearly labeled as sensitivity analyses and the grouped decompositions as group estimates based on China GBD shares rather than direct GBD outputs. Because the original hospital admission datasets used by the GBD Collaborators were not publicly available for re-estimation, case fatality was summarized only as an exploratory implied population-level fatality ratio, defined as estimated sepsis-related deaths divided by estimated incident sepsis cases, rather than as the original hospital-based CFR model.

## Results

Between 1990 and 2021, the absolute incidence of sepsis in China increased from 4,390,840 (95% UI: 3,373,824–5,522,713) to 6,773,717 million (95%UI: 4,828,778–9,275,569) (Table 1). This increase was observed in both females and males. The corresponding overall temporal patterns in absolute counts and crude rates are shown in Figure S1.

**Table 1:** Incidence of sepsis and sepsis-related mortality in China, 1990 and 2021

Sex	Incident cases		Death cases	
	1990	2021	1990	2021
Both sexes	4,390,840 (3,373,824–5,522,713)	6,773,717 (4,828,778–9,275,569)	2,795,000 (2,530,000–3,080,000)	2,480,000 (2,080,000–2,950,000)
Female	2,191,569 (1,691,112–2,745,641)	3,368,542 (2,412,199–4,595,774)	1,255,016 (1,102,494–1,421,181)	1,017,347 (817,663–1,288,756)
Male	2,199,271 (1,676,433–2,791,255)	3,405,175 (2,411,236–4,682,025)	1,539,984 (1,341,444–1,760,245)	1,462,653 (1,178,943–1,823,156)

Data are presented as *n* (95% Uncertainty interval).

Estimates for both sexes were aligned with the China-specific anchor estimates reported in the 1990 and 2021 publications. Female and male estimates were subsequently derived by applying model-based sex proportions to the corresponding combined-sex totals.

Over the same period, sepsis-related deaths decreased modestly from 2,795,000 (95% UI: 2,530,000–3,080,000) in 1990 to 2,480,000 (95% UI: 2,080,000–2,950,000) in 2021 (Table 1). Deaths declined in both females and males, although the male burden remained consistently higher. Supplementary Figure S1 further illustrates that the crude mortality trajectory declined before showing a rebound in the most recent period.

The overall implied fatality ratio of sepsis, defined as estimated sepsis-related deaths divided by estimated incident sepsis cases, declined from 63.7% (95% UI: 45.8–91.3) in 1990 to 36.6% (95% UI: 22.4–61.1) in 2021. Between 2019 and 2021, the overall implied fatality ratio remained broadly stable, changing from 36.7% (95% UI: 23.1–59.7) to 36.6% (95% UI: 22.4–61.1). We did not interpret cause-specific implied fatality ratios as clinical CFRs because the numerator and denominator were obtained from different model components and, in the group allocation based on China GBD shares, could become unstable. The corresponding exploratory implied fatality-ratio trends are shown in Supplementary Figure S2.

**Figure 1:**
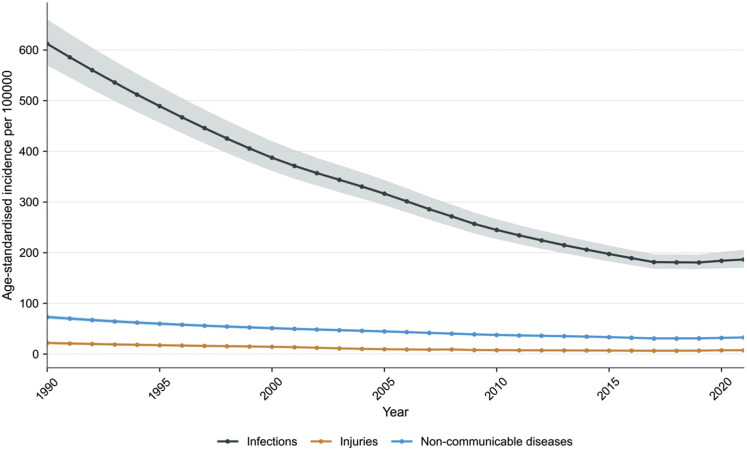
Age-standardized incidence of sepsis by broad underlying cause category in China, 1990–2021. Lines show estimated age-standardized incidence rates for infections, non-communicable diseases, and injuries; shaded areas indicate 95% uncertainty intervals.

Age-standardized incidence declined across all three broad underlying cause categories between 1990 and 2021 (Figure 1). In 2021, the highest age-standardized incidence rate was observed for infection-attributed sepsis, whereas non-communicable disease-attributed and injury-attributed incidence remained substantially lower throughout the study period. The corresponding age-standardized incidence and mortality trajectories are shown together in Supplementary Figure S3, and the sensitivity analysis for age-standardized incidence is presented in Supplementary Figure S4.

Further analysis of age-standardized rates in 2021 demonstrated clear differences by broad underlying cause category (Table 2). For both sexes combined, age-standardized incidence was highest for infections (186.5 per 100,000, 95% UI: 170.0–205.9), followed by non-communicable diseases (32.6 per 100,000, 95% UI: 30.5–35.2) and injuries (7.5 per 100,000, 95% UI: 6.5–8.7). Age-standardized mortality showed the same ordering, with infections accounting for the highest rate (26.6 per 100,000, 95% UI: 22.8–30.6), followed by non-communicable diseases (19.2 per 100,000, 95% UI: 16.3–22.2) and injuries (1.3 per 100,000, 95% UI: 1.1–1.5). Detailed 2021 count estimates by broad underlying cause category are summarized in Supplementary Table S2.

**Table 2:** Age-standardized incidence and mortality of sepsis according to broad underlying cause categories in China, 2021

Underlying cause category	Age-standardized incidence per 100,000 population (95% UI)			Age-standardized mortality per 100,000 population (95% UI)		
	Both sexes	Female	Male	Both sexes	Female	Male
Infections	186.5 (170.0–205.9)	182.8 (166.4–201.8)	190.1 (172.9–209.7)	26.6 (22.8–30.6)	19.9 (16.0–24.7)	36.4 (31.0–42.3)
Injuries	7.5 (6.5–8.7)	5.7 (5.0–6.6)	9.1 (7.9–10.7)	1.3 (1.1–1.5)	0.8 (0.7–1.0)	1.9 (1.5–2.2)
Non-communicable diseases	32.6 (30.5–35.2)	35.5 (33.3–38.2)	30.0 (27.9–32.4)	19.2 (16.3–22.2)	14.6 (12.0–17.9)	25.5 (21.0–31.0)

Data are presented as rate (95% UI).

UI: Uncertainty interval.

Cause-specific rates were estimated in sensitivity analyses for age-standardized incidence and mortality.

Sex disparities were evident across outcomes and cause categories. In 2021, males had substantially higher age-standardized mortality than females overall (63.4 per 100,000 *vs*. 35.3 per 100,000; Table 3) and across all three broad underlying cause categories (Table 2). For age-standardized incidence, males had higher rates than females for infection-attributed and injury-attributed sepsis, whereas females had slightly higher incidence for non-communicable disease-attributed sepsis.

**Table 3:** Age-standardized incidence and mortality of sepsis in China, 1990 and 2021

Sex	Age-standardized incidence per 100,000 population (95% UI)		Age-standardized mortality per 100,000 population (95% UI)	
	1990	2021	1990	2021
Both sexes	706.9 (657.1–763.5)	226.6 (207.0–249.8)	496.0 (449.2–541.5)	47.1 (40.3–54.4)
Female	727.9 (680.0–785.3)	230.6 (211.3–253.2)	431.1 (376.6–485.3)	35.3 (28.9–43.3)
Male	687.2 (634.0–747.7)	222.9 (202.8–246.5)	584.9 (517.2–656.2)	63.4 (52.8–75.7)

Data are presented as rate per 100,000 population (95% UI).

UI: Uncertainty interval.

These age-standardized rates were estimated in sensitivity analyses.

**Figure 2:**
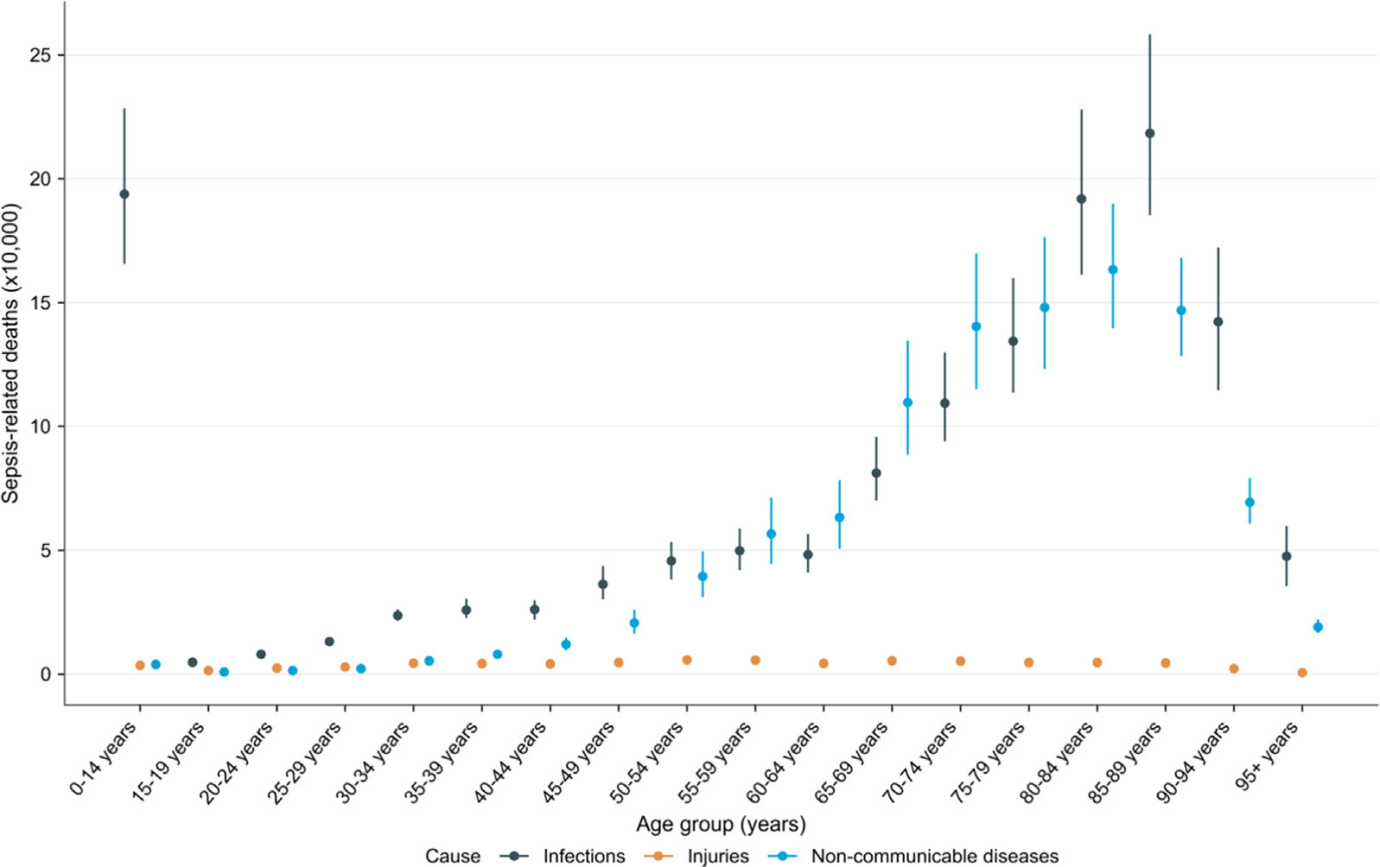
Sepsis-related deaths by broad underlying cause category and age group in China, 2021. Bars show estimated death counts by age group for infections, non-communicable diseases, and injuries; error bars indicate 95% uncertainty intervals.

Analysis of age-specific sepsis-related death counts in 2021 highlighted distinct burden patterns (Figure 2). Infection-related sepsis deaths showed substantial burdens in both children and older adults, with the highest counts observed at age 85–89 years; injury-related deaths remained comparatively low across all age groups, whereas non-communicable disease-related sepsis deaths rose sharply with age and peaked at 80–84 years. These age-structured patterns complement the age-standardized analyses shown in Figure 1 and Figures S3–S5.

Further analyses clarified these temporal patterns. Cause-group decomposition showed that infections and non-communicable diseases consistently dominated the estimated burden, whereas injuries contributed only a small fraction across the full study period (Supplementary Figure S6). In the mortality scope analysis, infectious underlying causes remained the leading contributor to sepsis-related deaths, accounting for 66.5% in 1990 and 56.5% in 2021, whereas the contribution of non-infectious causes increased over time (Supplementary Figure S7). In the age-standardized sensitivity analyses, both incidence and mortality declined through 2019, followed by a modest rebound or plateau in 2020–2021 (Supplementary Figures S4 and S5). Alternative interpolation approaches yielded nearly identical 2020 national estimates, indicating that the main pandemic-period interpretation was not driven by the choice of interpolation method (Supplementary Table S1).

## Discussion

Sepsis remains a major source of health loss in China. Although age-standardized incidence and mortality declined over time, the absolute burden remained high. Infectious causes still accounted for the largest share of mortality, while chronic disease and population ageing increasingly shaped vulnerability at the population level. Reducing preventable sepsis deaths in China will therefore require a combined strategy that strengthens infection prevention and early sepsis care while embedding sepsis prevention within chronic disease management and healthy ageing programs.

This study has several strengths. By applying a consistent GBD-based framework across 1990–2021, we were able to examine long-term sepsis trends across infections, injuries, and non-communicable diseases (NCDs) within a single analytical structure. That approach also allowed us to consider sepsis as an intermediate condition across a broad range of underlying causes rather than limiting interpretation to hospitalized cases alone. The study also has important limitations. First, the analysis inherits uncertainty from GBD estimates and remains vulnerable to variation in coding practice, underreporting, misclassification, data availability, and the recording of sepsis as an intermediate rather than underlying cause of death. Second, the age-standardized sensitivity analyses rely on China GBD age-standardized trajectories through 2019, with the 2019–2021 crude-rate change applied thereafter; this may understate or overstate the true age-standardized response during the COVID-19 period if the age structure of sepsis changed differently from the crude national trend or if pandemic-related changes in infection patterns and healthcare use were not fully captured by crude national rates. Third, the exploratory implied fatality ratio should not be interpreted as a hospital-based case fatality ratio because deaths and incidence were estimated from different model components, time lags were not modeled, and group allocation based on China GBD shares can yield unstable cause-specific values. Fourth, although piecewise log-linear interpolation was used as the main approach, sensitivity analyses with linear and monotonic cubic Hermite interpolation produced the same qualitative conclusions and very similar 2020 estimates, with the largest divergence occurring in the pre-2019 interpolated segment rather than during the pandemic years. Finally, we could not directly validate coding algorithms, estimate hospital-based case fatality, or examine province-specific hospital tiers because the original individual-level inputs were not publicly available.

These findings have direct implications for prevention and health policy in China. Infection prevention should remain a central priority because infection-attributed sepsis continues to account for the largest mortality burden despite declining incidence. Continued investment is needed in vaccination, infection control, antimicrobial stewardship, rapid recognition of severe infection, and timely escalation of care. At the same time, the rising contribution of NCD-attributed sepsis suggests that sepsis prevention should be integrated into chronic disease management, especially for patients with diabetes, chronic cardiopulmonary disease, chronic kidney disease, malignancy, frailty, and multimorbidity.^[^^12^^]^ The persistent male excess further suggests that prevention and early-recognition strategies should be tailored to high-risk male populations, especially those with heavy smoking exposure, occupational risk, and delayed healthcare utilization. Age-specific prevention pathways are also needed, including stronger infection prevention in children and more proactive surveillance for deterioration in older adults.

The age-specific patterns support this interpretation. Infection-attributed sepsis remained important in children, whereas NCD-attributed mortality rose sharply with age and became the dominant component in older adults. This distribution is clinically plausible. Children remain vulnerable because of developmental susceptibility to severe infection, while older adults are increasingly affected by frailty, multimorbidity, immunosenescence, and repeated exposure to healthcare settings.^[^[13], [14], [15], [16]^]^ That pattern is also consistent with broader evidence showing that pediatric and neonatal sepsis remain clinically important outside China.^[^^17^^,^^18^^]^ The shift toward NCD-attributed sepsis in later life matters for health-system planning because future reductions in sepsis mortality will depend not only on better acute infection management but also on stronger longitudinal management of high-risk older adults with complex chronic disease.

The persistent male disadvantage also warrants explicit interpretation. Across infection-, injury-, and NCD-attributed sepsis, males had consistently higher age-standardized incidence and mortality than females. This pattern has also been reported in previous Chinese studies.^[^^8^^,^^15^^,^^19^^]^ Several explanations are plausible. Chinese men have historically had much higher smoking exposure than women, which may increase the risk of respiratory infection, chronic cardiopulmonary disease, and adverse outcomes after severe infection.^[^^14^^]^ Men may also face greater exposure to occupational hazards, injuries, and some chronic cardiometabolic conditions, which could widen the male-female gap in both infection- and NCD-attributed mortality. Biological differences in immune and hormonal responses, together with delayed healthcare seeking or later presentation, may further worsen outcomes once infection occurs. Although the present analysis cannot disentangle the relative contribution of these mechanisms, the consistency of the male excess across multiple Chinese datasets suggests that sex disparity is a stable epidemiological feature of sepsis in China and deserves targeted preventive and clinical attention.

Our findings are broadly consistent with previous studies from China while extending the current literature. The nationwide analysis of hospitalized sepsis by Weng and colleagues showed that the burden remained concentrated in males and older adults and that in-hospital case fatality, although declining, remained clinically important.^[^^8^^]^ Earlier work on sepsis-related mortality also documented a substantial national burden with marked age and sex variation.^[^^16^^]^ A more recent population-based analysis showed that sepsis-related mortality changed over time but remained a major public health problem.^[^^19^^]^ Systematic reviews and meta-analyses from China have likewise reported a high prevalence of sepsis and persistently high mortality in sepsis and septic shock.^[^^6^^,^^7^^]^ Comparable epidemiological studies from the United States, Australia and New Zealand, Spain, and Brazil have also shown that sepsis remains a substantial burden across diverse health systems, although case definitions and data sources differ.^[^^2^^,^^3^^,^^20^^,^^21^^]^ The present study adds to this evidence by examining underlying-cause attribution over 3 decades and by showing that, within China's epidemiological transition, infection-attributed sepsis still dominates mortality whereas NCD-attributed sepsis is becoming increasingly prominent.

The growing contribution of NCD-attributed sepsis most likely reflects China's broader epidemiological transition. Chronic conditions such as diabetes, cardiovascular and cerebrovascular disease, chronic kidney disease, chronic pulmonary disease, and malignancy have become increasingly common over recent decades, particularly in middle-aged and older adults.^[^^15^^,^^22^^]^ These conditions increase susceptibility to infection through several pathways, including immune dysfunction, repeated healthcare contact, invasive procedures, device use, and recurrent hospitalization. They also reduce physiological reserve, making it more likely that an acute infectious insult will progress to organ failure. In this context, the rise in NCD-attributed sepsis should not be interpreted as separate from infection-related pathology. Rather, it points to a growing population in whom chronic disease amplifies the severity and lethality of infectious episodes.

The divergence between infection-attributed and NCD-attributed sepsis is biologically and epidemiologically plausible in the Chinese setting. The decline in infection-attributed incidence probably reflects long-term improvements in sanitation, vaccine uptake, maternal and child healthcare, and control of common communicable diseases. Even so, infection-attributed sepsis remained the largest contributor to mortality, suggesting that severe infection still carries high lethality once organ dysfunction develops. That interpretation is consistent with previous Chinese studies showing that sepsis-related mortality remains substantial at the national level and that temporal reductions in mortality have been incomplete.^[^^8^^,^^16^^,^^19^^]^ It also aligns with recent China-wide burden analyses showing that infection-related mortality remains high in vulnerable age groups and continues to represent a major national health challenge.^[^^9^^]^ In practical terms, progress in primary infection prevention has not yet translated into an equally large reduction in deaths among patients who progress to severe sepsis.

This study provides a population-level assessment of sepsis incidence and mortality in China from 1990 to 2021 and clarifies how the epidemiological context of sepsis has changed over time. Four findings deserve emphasis. First, age-standardized incidence and mortality declined, yet the absolute burden remained high. Second, infection-attributed sepsis remained the leading contributor to sepsis-related mortality in 2021. Third, the contribution of NCD-attributed sepsis increased over time, indicating that chronic disease is becoming more important in shaping sepsis risk in China. Fourth, the burden remained higher in males than in females and became increasingly concentrated in older adults for NCD-attributed sepsis. Taken together, these findings suggest that sepsis in China is increasingly shaped by the interaction between severe infection, population ageing, multimorbidity, and unequal risk exposure across demographic groups.

## CRediT authorship contribution statement

**Mingmin Pang:** Visualization, Software, Formal analysis, Data curation. **Qi Zhang:** Writing – review & editing, Methodology, Investigation. **Haoyu Wang:** Writing – original draft, Methodology, Formal analysis. **Shihan Zhang:** Software, Resources, Formal analysis. **Yanan Li:** Validation, Data curation. **Mengfei Lu:** Visualization, Validation, Methodology. **Miaosa Zhang:** Visualization, Software. **Hao Wang:** Writing – review & editing, Supervision, Funding acquisition.

## Appendix Supplementary materials

Image, application 1
