# Supplementary material for: Sepsis incidence and mortality in China, 1990–2021: An analysis for the Global Burden of Disease Study
Source: J Intensive Med. 2026 May 6;6(4):351–7. doi: 10.1016/j.jointm.2026.04.004 (PMC13323530; doi:10.1016/j.jointm.2026.04.004)
Supplement: Supplementary file 1 [file mmc1.docx]

**Table S1. Sensitivity analysis comparing alternative interpolation methods for the national reconstructed series**

Data are 2020 values under each interpolation method and the maximum absolute percentage difference from the piecewise log-linear series over 1990?2021. PCHIP=piecewise cubic Hermite interpolating polynomial.

| Outcome | 2020 log-linear | 2020 linear | 2020 PCHIP | Max abs diff vs log-linear (%) |
| --- | --- | --- | --- | --- |
| Incident sepsis cases, n | 6,626,833 | 6,628,426 | 6,617,636 | 5.62 |
| Incidence rate per 100,000 | 467.2 | 467.2 | 466.2 | 4.06 |
| Sepsis-related deaths, n | 2,429,486 | 2,430,000 | 2,416,463 | 5.81 |
| Mortality rate per 100,000 | 171.2 | 171.2 | 170.4 | 11.57 |

Interpolation sensitivity analyses using piecewise linear and monotonic cubic Hermite interpolation preserved the same qualitative conclusions as the main piecewise log-linear reconstruction. The 2020 estimates were nearly identical across methods, and the largest deviations were confined to the pre-2019 interpolated segment (Supplementary Table S1). This supports the robustness of the pandemic-period interpretation while showing that mid-period annual levels should still be understood as reconstructed rather than directly observed values.

**Table S2.** Incident sepsis cases and sepsis-related deaths by broad underlying cause category in China, 2021

Data are n (95% UI). UI=uncertainty interval. Estimates were derived from the recalibrated 2025 paper-aligned China series.

| Underlying cause category | Incident sepsis cases, n (95% UI) | Sepsis-related deaths, n (95% UI) |
| --- | --- | --- |
| Infections | 5,574,797 (3,966,044-7,644,361) | 1,400,000 (1,180,000-1,660,000) |
| Injuries | 223,539 (152,128-322,490) | 69,954 (58,295-83,556) |
| Non-communicable diseases | 975,381 (710,606-1,308,717) | 1,010,046 (841,705-1,206,444) |

**Table S3.** Explicit ICD-10-CM codes for sepsis

| ICD-10-CM | Codes Description^a^ |
| --- | --- |
| A01.003 | Typhoid fever sepsis |
| A02.100 | Salmonella sepsis |
| A03.900 | Shigellosis, unspecified (shock) |
| A09.005 | Septic gastroenteritis |
| A20.7 | Septicemic plague |
| A21.7 | Generalized tularemia |
| A22.7 | Anthrax sepsis |
| A24.1 | Acute and fulminating melioidosis |
| A26.7 | Erysipelothrix sepsis |
| A27.900 | Leptospirosis |
| A28.001 | Pasteurellosis |
| A28.2 | Extraintestinal yersiniosis |
| A32.7 | Listerial sepsis |
| A38.x00x012 | Scarlet fever sepsis |
| A39.2 | Acute meningococcemia |
| A39.3 | Chronic meningococcemia |
| A39.4 | Meningococcemia， unspecified |
| A39.1 | Waterhouse |
| A40 | Streptococcal sepsis |
| A41 | Other sepsis |
| A42.7 | Actinomycotic sepsis |
| A48.3 | Toxic shock syndrome |
| A54.8 | Other gonococcal infections (sepsis) |
| A93.800x001 | Other specified arthropod-borne viral fevers [Piry virus disease] |
| A98.500 | Haemorrhagic fever with renal syndrome |
| B00.7 | Herpetic septicemia |
| B37.7 | Candidal sepsis |
| B37.6 | Candidal endocarditis |
| B49 | Unspecified mycosis (Fungemia) |
| F05.901 | Delirium, unspecified (infectious) |
| F06.800x002 | Other specified mental disorders due to brain damage and dysfunction and to physical disease (biliary infection) |
| F06.800x016 | Other specified mental disorders due to brain damage and dysfunction and to physical disease (bacillary dysentery) |
| J15.903 | Bacterial pneumonia, unspecified (severe community-acquired) |
| J18.903 | Pneumonia, unspecified (severe) |
| J95.000x001 | Sepsis of tracheostomy stoma |
| K85.800x019 | Abscess of pancreas (severe) |
| K85.817 | Abscess of pancreas (other, severe) |
| O03.300x001 | Spontaneous abortion, Incomplete, with septic shock |
| O03.800x001 | Spontaneous abortion, Complete, with septic shock |
| O04.300x004 | Medical abortion, Incomplete, with septic shock |
| O04.800x001 | Medical abortion, Complete, with septic shock |
| O04.804 | Medical abortion, later complete, with septic shock |
| O08.000 | Genital tract and pelvic infection following abortion and ectopic and molar pregnancy |
| O08.003 | Septic shock following abortion and ectopic and molar pregnancy |
| O08.200x002 | Embolism following abortion and ectopic and molar pregnancy (septic) |
| O08.200x006 | Embolism following abortion and ectopic and molar pregnancy (septicopyaemic) |
| O75.3 | Other infection during labour |
| O85 | Puerperal sepsis |
| O88.300 | Obstetric pyaemic and septic embolism |
| O98.8 | Other maternal infectious and parasitic diseases complicating pregnancy, childbirth and the puerperium |
| P36 | Bacterial sepsis of newborn |
| P37.800x002 | Other specified congenital infectious and parasitic diseases |
| R57.2 | Septic shock |
| R65.1 | Systemic inflammatory response syndrome (SIRS) due to infection with organ dysfunction |
| T80.2 | Infections following infusion, transfusion and therapeutic injection |
| T81.4 | Infection following a procedure, not elsewhere classified |
| T88.000x002 | Sepsis following immunization |

a Any diagnosis with the word "severe" required complications of organ dysfunction in the official 10-digit Chinese version of the International Classification of Diseases, tenth revision (ICD-10).

**Table S4**. ICD-9-CM and ICD-10-CM codes used to identify infection in NDCMS and sepsis-related death in NMSS

| ICD-9-CM | Codes Description | ICD-10-CM |
| --- | --- | --- |
| 001 | Cholera | A00 |
| 002 | Typhoid/paratyphoid fever | A01 |
| 003 | Other salmonella infection | A02  G01  J17  M01  M90 |
| 004 | Shigellosis | A03 |
| 005 | Other food poisoning | A05 |
| 008 | Intestinal infection not otherwise classified | A02  A04  A08 |
| 009 | Ill-defined intestinal infection | A09 |
| 010 | Primary tuberculosis | A15  A16 |
| 011 | Pulmonary tuberculosis | A15  A16 |
| 012 | Other respiratory tuberculosis | A15  A16  J38 |
| 013 | Central nervous system tuberculosis | A17 |
| 014 | Intestinal tuberculosis | A18  K93 |
| 015 | Tuberculosis of bone and joint | A18  H75 |
| 016 | Genitourinary tuberculosis | A18  N51  N74 |
| 017 | Tuberculosis not otherwise classified | A18  D77  E35  K23 |
| 018 | Military tuberculosis | A19 |
| 020 | Plague | A20 |
| 021 | Tularemia | A21 |
| 022 | Anthrax | A22 |
| 023 | Brucellosis | A23 |
| 024 | Glanders | A24 |
| 025 | Melioidosis | A24 |
| 026 | Rat-bite fever | A25 |
| 027 | Other bacterial zoonoses | A26  A28  A32 |
| 030 | Leprosy | A30 |
| 031 | Other mycobacterial disease | A31 |
| 032 | Diphtheria | A36  I41  K67  N33 |
| 033 | Whooping cough | A37 |
| 034 | Streptococcal throat/scarlet fever | A38  J02 |
| 035 | Erysipelas | A46 |
| 036 | Meningococcal infection | A39  G05  H48  M01 |
| 037 | Tetanus | A35 |
| 038 | Septicemia | A40  A41 |
| 039 | Actinomycotic infections | A42  A43  B47 |
| 040 | Other bacterial diseases | A48  K90  M60 |
| 041 | Bacterial infection in other diseases not otherwise specified | B95  B96 |
| 090 | Congenital syphilis | A50 |
| 091 | Early symptomatic syphilis | A51  R59  H22  H32  M90  K77  G01  L99 |
| 092 | Early syphilis latent | A51 |
| 093 | Cardiovascular syphilis | A52  I32  I39  I41  I79 |
| 094 | Neurosyphilis | A52  G01  G05  H32  H48  H94  I60 |
| 095 | Other late symptomatic syphilis | A52  H19  J99  K67  K77  N29  M63  M68  M90 |
| 096 | Late syphilis latent | A52 |
| 097 | Other and unspecified syphilis | A52  A53 |
| 098 | Gonococcal infections | A54  N30  N33  N39  N51  N72  N74  H19  G01  I32  I39  I52  K67 |
| 100 | Leptospirosis | A27  G01 |
| 101 | Vincent’s angina | A69 |
| 102 | Yaws | A66 |
| 103 | Pinta | A67 |
| 104 | Other spirochetal infection | A65  A69 |
| 110 | Dermatophytosis | B35 |
| 111 | Dermatomycosis not otherwise classified or specified | B36 |
| 112 | Candidiasis | A09  B37  H60 |
| 114 | Coccidioidomycosis | B38 |
| 115 | Histoplasmosis | B39  G02  H36  I32  I39  J99 |
| 116 | Blastomycotic infection | B40  B41  B48 |
| 117 | Other mycoses | B42  B43  B44  B45  B46  B47  B48 |
| 118 | Opportunistic mycoses | B48 |
| 320 | Bacterial meningitis | G00 |
| 322 | Meningitis, unspecified | G03  G05 |
| 324 | Central nervous system abscess | G06 |
| 325 | Phlebitis of intracranial sinus | G08 |
| 420 | Acute pericarditis | I30  I32 |
| 421 | Acute or subacute endocarditis | I33  I39 |
| 451 | Thrombophlebitis | I80 |
| 461 | Acute sinusitis | J01 |
| 462 | Acute pharyngitis | J02 |
| 463 | Acute tonsillitis | J03 |
| 464 | Acute laryngitis/tracheitis | J04  J05 |
| 465 | Acute upper respiratory infection of multiple sites/not otherwise specified | J06 |
| 481 | Pneumococcal pneumonia | J13 |
| 482 | Other bacterial pneumonia | J14  J15 |
| 485 | Bronchopneumonia with organism not otherwise specified | J18 |
| 486 | Pneumonia, organism not otherwise specified | J18 |
| 491.21 | Acute exacerbation of obstructive chronic bronchitis | J44.1 |
| 494 | Bronchiectasis | J47 |
| 510 | Empyema | J86 |
| 513 | Lung/mediastinum abscess | J85 |
| 540 | Acute appendicitis | K35 |
| 541 | Appendicitis not otherwise specified | K37 |
| 542 | Other appendicitis | K36 |
| 562.01 | Diverticulitis of small intestine without hemorrhage | K57.12 |
| 562.03 | Diverticulitis of small intestine with hemorrhage | K57.13 |
| 562.11 | Diverticulitis of colon without hemorrhage | K57.22 |
| 562.13 | Diverticulitis of colon with hemorrhage | K57.23 |
| 566 | Anal and rectal abscess | K61 |
| 567 | Peritonitis | K65  K67 |
| 569.5 | Intestinal abscess | K63.0 |
| 569.83 | Perforation of intestine | K63.1 |
| 572.0 | Abscess of liver | K75.0 |
| 572.1 | Portal pyema | K75.1 |
| 575.0 | Acute cholecystitis | K81.0 |
| 590 | Kidney infection | N10  N11  N12  N15 |
| 597 | Urethritis/urethral syndrome | N34 |
| 599.0 | Urinary tract infection not otherwise specified | N39.0 |
| 601 | Prostatic inflammation | N41  N51 |
| 614 | Female pelvic inflammation disease | N70  N73 |
| 615 | Uterine inflammation disease | N71 |
| 616 | Other female genital inflammation | N72  N75  N76  N77 |
| 681 | Cellulitis, finger/toe | L03 |
| 682 | Other cellulitis or abscess | L03 |
| 683 | Acute lymphadenitis | L04 |
| 686 | Other local skin infection | L08  L98 |
| 711.0 | Pyogenic arthritis | M00.9 |
| 730 | Osteomyelitis | M86  M89  M90 |
| 790.7 | Bacteremia | A49.9 |
| 996.6 | Infection or inflammation of device/graft | T82.6  T82.7  T83.5  T84.5  T84.6  T85.71  T85.78  T85.81 |
| 998.5 | Postoperative infection | T81.4 |
| 999.3 | Infectious complication of medical care not otherwise classified | T80.2 |
| Extra codes | |  |
|  | Bacterial infection of unspecified site | A49 |
|  | Typhus fever | A75 |
|  | Spotted fever [tick-borne rickettsioses] | A77 |
|  | Q fever | A78 |
|  | Other rickettsioses | A79 |
|  | Acute poliomyelitis | A80 |
|  | Atypical virus infections of central nervous system | A81 |
|  | Rabies | A82 |
|  | Mosquito-borne viral encephalitis | A83 |
|  | Tick-borne viral encephalitis | A84 |
|  | Other viral encephalitis, not elsewhere classified | A85 |
|  | Unspecified viral encephalitis | A86 |
|  | Viral meningitis | A87 |
|  | Other viral infections of central nervous system, not elsewhere classified | A88 |
|  | Unspecified viral infection of central nervous system | A89 |
|  | Dengue fever [classical dengue] | A90 |
|  | Dengue hemorrhagic fever | A91 |
|  | Other mosquito-borne viral fevers | A92 |
|  | Other arthropod-borne viral fevers, not elsewhere classified | A93 |
|  | Unspecified arthropod-borne viral fever | A94 |
|  | Yellow fever | A95 |
|  | Arenaviral hemorrhagic fever | A96 |
|  | Other viral hemorrhagic fevers, not elsewhere classified | A98 |
|  | Unspecified viral hemorrhagic fever | A99 |
|  | Other viral diseases, not elsewhere classified | B33 |
|  | Viral infection of unspecified site | B34 |
|  | Unspecified mycosis | B49 |
|  | Plasmodium falciparum malaria | B50 |
|  | Plasmodium vivax malaria | B51 |
|  | Plasmodium malariae malaria | B52 |
|  | Other specified malaria | B53 |
|  | Unspecified malaria | B54 |
|  | Leishmaniasis | B55 |
|  | African trypanosomiasis | B56 |
|  | Chagas' disease | B57 |
|  | Toxoplasmosis | B58 |
|  | Pneumocystosis | B59 |
|  | Other protozoal diseases, not elsewhere classified | B60 |
|  | Unspecified protozoal disease | B64 |
|  | Schistosomiasis [bilharziasis] | B65 |
|  | Other fluke infections | B66 |
|  | Echinococcosis | B67 |
|  | Taeniasis | B68 |
|  | Cysticercosis | B69 |
|  | Diphyllobothriasis and sparganosis | B70 |
|  | Other cestode infections | B71 |
|  | Dracunculiasis | B72 |
|  | Onchocerciasis | B73 |
|  | Filariasis | B74 |
|  | Trichinellosis | B75 |
|  | Hookworm diseases | B76 |
|  | Ascariasis | B77 |
|  | Strongyloidiasis | B78 |
|  | Trichuriasis | B79 |
|  | Enterobiasis | B80 |
|  | Other intestinal helminthiases, not elsewhere classified | B81 |
|  | Unspecified intestinal parasitism | B82 |
|  | Other helminthiases | B83 |
|  | Pediculosis and phthiriasis | B85 |
|  | Scabies | B86 |
|  | Myiasis | B87 |
|  | Other infestations | B88 |
|  | Unspecified parasitic disease | B89 |
|  | Sequelae of tuberculosis | B90 |

NDCMS=National Data Center for Medical Service. NMSS=National Mortality Surveillance System.

**Table S5.** ICD-10-CM codes for organ dysfunction

| System | ICD-10-CM | Codes Description |
| --- | --- | --- |
| Cardiovascular | A41.9 | Septic shock |
|  | A48.3 | Toxic shock syndrome |
|  | E86.x00 | Volume depletion |
|  | E86.x00x001 | hypovolemia |
|  | E86.x00x003 | extracellular fluid deletion |
|  | E86.x00x004 | Plasma volume depletion |
|  | E86.x00x005 | Volume depletion |
|  | E86.x01 | dehydration |
|  | I51.400x007 | Severe Myocarditis |
|  | I95.8 | Hypotension, unspecified |
|  | I95.9 | Hypotension, |
|  | I99.x00 | Other disorders of the circulatory system |
|  | I99.x01 | Circulatory disorders |
|  | R09.800x082 | Weak pulse |
|  | R57.0 | Cardiogenic shock |
|  | R57.1 | Hypovolaemic shock |
|  | R57.2 | Septic shock |
|  | R57.8 | Other shock |
|  | R57.9 | Shock, unspecified |
|  | O03.300x001 | Spontaneous abortion, Incomplete, with septic shock |
|  | O03.800x001 | Spontaneous abortion, Complete, with septic shock |
|  | O04.300x004 | Medical abortion, Incomplete, with septic shock |
|  | O04.800x001 | Medical abortion, Complete, with septic shock |
|  | O04.804 | Medical abortion, later complete, with septic shock |
|  | P29 | Cardiovascular disorders originating in the perinatal period |
|  |  |  |
| Respiratory | J80 | Acute respiratory distress syndrome |
|  | J81 | Pulmonary oedema |
|  | J95.100 | Acute pulmonary insufficiency after thoracic surgery |
|  | J95.200 | Acute pulmonary insufficiency after non-thoracic surgery |
|  | J95.800x004 | Respiratory failure after surgical procedures |
|  | J95.800x021 | Respiratory distress syndrome in adults after surgery |
|  | J96 | Acute respiratory failure with hypoxia, not elsewhere classified |
|  | J96.9 | Respiratory failure, unspecified with hypoxia |
|  | J98.4 | Other disorders of lung |
|  | J98.400x024 | Severe infection of the lungs |
|  | P22 | Respiratory distress of newborn |
|  | P28.5 | Respiratory failure of newborn |
|  | R09.0 | Asphyxia |
|  | R09.000 | suffocate |
|  | R09.2 | Respiratory arrest |
|  | R09.800x095 | Asthma |
|  | U04.9 | Severe acute respiratory syndrome [SARS], unspecified |
|  |  |  |
| Central nervous system | F05 | Delirium (not alcohol- or drug-induced) |
|  | F06.8 | Other specified mental disorders due to infection |
|  | G93.1 | Anoxic brain damage, not elsewhere classified |
|  | G93.4 | Encephalopathy, unspecified |
|  | G93.8 | Other specified disorders of brain |
|  | G93.9 | Disorder of brain, unspecified |
|  | R40 | Somnolence, stupor and coma |
|  | R41.0 | Disorientation, unspecified |
|  | R45.3 | Demoralization and apathy |
|  | R55 | Syncope and collapse |
|  |  |  |
| Renal | A98.500 | Haemorrhagic fever with renal syndrome |
|  | N17 | Acute kidney failure |
|  | N19 | Unspecified kidney failure |
|  | R34 | Anuria and oliguria |
|  | R39.2 | Extrarenal uraemia |
|  | R94.4 | Abnormal results of kidney function studies |
|  |  |  |
| Metabolic | E87.2 | Acidosis |
|  |  |  |
| Hematologic | A93.800x001 | Other specified arthropod-borne viral fevers [Piry virus disease] |
|  | D61.900x001 | Bone marrow suppression |
|  | D61.901 | Myelosuppressive anemia |
|  | D61.903 | Pancytopenia |
|  | D61.906 | Acute bone marrow hematopoietic function inhibition |
|  | D65 | Disseminated intravascular coagulation [defibrination syndrome] |
|  | D65.x00x003 | Gangrene purpura |
|  | D65.x01 | Acquired fibrinogen deficiency |
|  | D65.x02 | Acquired fibrinolytic bleeding |
|  | D65.x03 | Fibrinolytic purpura |
|  | D68.9 | Coagulation defect, unspecified |
|  | D69.000x008 | Infectious purpura |
|  | D69.000x011 | Bacterial purpura |
|  | D69.000x013 | Toxic purpura |
|  | D69.203 | purpura |
|  | D69.301 | Hemorrhagic purpura |
|  | D69.5 | Secondary thrombocytopenia |
|  | D69.501 | Secondary thrombocytopenic purpura |
|  | D69.6 | Thrombocytopenia, unspecified |
|  | D69.8 | Other specified haemorrhagic conditions |
|  | D76.200x001 | Infectious hemophagocytic syndrome |
|  | D76.200x011 | Infectious erythrophagocytic syndrome |
|  |  |  |
| Hepatic | B15.000 | Hepatitis A, accompanied by hepatic coma |
|  | B15.001 | Acute viral hepatitis A with hepatic coma |
|  | B15.002 | Acute severe severe viral hepatitis A with hepatic coma |
|  | B15.003 | Subacute severe viral hepatitis A with hepatic coma |
|  | B16.000 | Acute hepatitis B, with δ factor (co-infection) and accompanied by hepatic coma |
|  | B16.001 | Acute hepatitis B-D with hepatic coma |
|  | B16.200 | Acute hepatitis B, not accompanied by δ factor (co-infection), but with hepatic coma |
|  | B16.201 | Acute viral hepatitis B with hepatic coma |
|  | B16.202 | Subacute severe viral hepatitis B with hepatic coma |
|  | B16.203 | Acute severe hepatitis B with hepatic coma |
|  | B16.204 | Acute jaundice-free hepatitis B with hepatic coma |
|  | B16.206 | Acute severe hepatitis B with hepatic coma |
|  | B17.807 | Acute severe hepatitis hepatitis |
|  | B19.000 | Viral hepatitis, accompanied by hepatic coma |
|  | B19.000x001 | Viral hepatitis with hepatic coma |
|  | B19.001 | Acute severe viral hepatitis with hepatic coma |
|  | B19.002 | Subacute severe viral hepatitis with hepatic coma |
|  | B25.101† | Cytome hepatitis with hepatic coma |
|  | E80.600 | Bilirubin metabolism disorders, others |
|  | E80.604 | Hyperbilirubinemia |
|  | E80.700 | Bilirubin metabolism disorders |
|  | K71.100x001 | Toxic liver disease with liver failure |
|  | K71.103 | Toxic liver failure |
|  | K72.0 | Acute and subacute hepatic failure |
|  | K72.9 | Hepatic failure, unspecified |
|  | K76.7 | Hepatorenal syndrome |
|  | K76.8 | Other specified diseases of liver |
|  | K76.9 | Liver disease, unspecified |
|  | K91.825 | Liver failure after surgery |
|  |  |  |
| Others | A01.003 | Typhoid fever sepsis |
|  | A02.100 | Salmonella sepsis |
|  | A03.900 | Shigellosis, unspecified (shock) |
|  | A09.005 | Septic gastroenteritis |
|  | A20.7 | Septicemic plague |
|  | A21.7 | Generalized tularemia |
|  | A22.7 | Anthrax sepsis |
|  | A24.1 | Acute and fulminating melioidosis |
|  | A26.7 | Erysipelothrix sepsis |
|  | A27.900 | Leptospirosis |
|  | A28.001 | Pasteurellosis |
|  | A28.2 | Extraintestinal yersiniosis |
|  | A32.7 | Listerial sepsis |
|  | A38.x00x012 | Scarlet fever sepsis |
|  | A39.2 | Acute meningococcemia |
|  | A39.3 | Chronic meningococcemia |
|  | A39.4 | Meningococcemia， unspecified |
|  | A39.1 | Waterhouse |
|  | A40. | Streptococcal sepsis |
|  | A41. | Other sepsis |
|  | A42.7 | Actinomycotic sepsis |
|  | A49.103 | Streptococcal infection syndrome |
|  | A54.8 | Other gonococcal infections (sepsis) |
|  | A88.800x001 | Polio-like syndrome |
|  | B00.7 | Herpetic septicemia |
|  | B37.7 | Candidal sepsis |
|  | B37.6 | Candidal endocarditis |
|  | B49 | Unspecified mycosis (Fungemia) |
|  | D71.x00x005 | Progressive septic granulomatous disease |
|  | J15.903 | Bacterial pneumonia, unspecified (severe community-acquired) |
|  | J18.903 | Pneumonia, unspecified (severe) |
|  | J95.000x001 | Sepsis of tracheostomy stoma |
|  | O08.000x006 | Sepsis following abortion and ectopic and molar pregnancy |
|  | O08.200x002 | Embolism following abortion and ectopic and molar pregnancy (septic) |
|  | O08.200x006 | Embolism following abortion and ectopic and molar pregnancy (septicopyaemic) |
|  | O85 | Puerperal sepsis |
|  | O88.300 | Obstetric pyaemic and septic embolism |
|  | P36 | Bacterial sepsis of newborn |
|  | P37.800x002 | Other specified congenital infectious and parasitic diseases |
|  | R09.800 | Involves other specific signs and symptoms of the circulatory and respiratory systems |
|  | R65.1 | Systemic inflammatory response syndrome (SIRS) due to infection with organ dysfunction |
|  | T80.2 | Infections following infusion, transfusion and therapeutic injection |
|  | T81.4 | Infection following a procedure, not elsewhere classified |
|  | T88.000x002 | Sepsis following immunization |
|  |  |  |
| Multiple | R68.800x001 | Multiple organ failure |

**Table S6.** ICD-9-CM codes for organ dysfunction-related procedures

| Organ | ICD-9-CM | Codes Description |
| --- | --- | --- |
| Respiratory | 311 | Temporary tracheostomy |
| Cardiovascular | 3129 | Other permanent tracheostomy |
| Cardiovascular | 3893 | Venous catheterization, not elsewhere classified |
| Renal | 3895 | Venous catheterization for renal dialysis |
| Cardiovascular | 3897 | Central venous catheter placement with guidance |
| Cardiovascular | 3899 | Other puncture of vein |
| Renal | 3927 | Arteriovenostomy for renal dialysis |
| Renal | 3942 | Revision of arteriovenous shunt for renal dialysis |
| Others | 3965 | Extracorporeal membrane oxygenation [ECMO] |
| Renal | 3995 | Hemodialysis |
| Hepatic | 5092 | Extracorporeal hepatic assistance |
| Renal | 5498 | Peritoneal dialysis |
| Respiratory | 9390 | Non-invasive mechanical ventilation |
| Respiratory | 9391 | Intermittent positive pressure breathing [IPPB] |
| Others | 9393 | Nonmechanical methods of resuscitation |
| Respiratory | 9604 | Insertion of endotracheal tube |
| Respiratory | 9670 | Continuous invasive mechanical ventilation of unspecified duration |
| Respiratory | 9671 | Continuous invasive mechanical ventilation for less than 96 consecutive hours |
| Respiratory | 9672 | Continuous invasive mechanical ventilation for 96 consecutive hours or more |
| Others | 9960 | Cardiopulmonary resuscitation, not otherwise specified |
| Others | 9962 | Other electric countershock of heart |
| Others | 9963 | Closed chest cardiac massage |

**Table S7.** Hierarchical grouping of underlying causes of death

| **GBD cause** | **Nesting group** |
| --- | --- |
| HIV/AIDS | Infectious (b) |
| HIV/AIDS - Drug-susceptible tuberculosis | Infectious (b) |
| HIV/AIDS - Multidrug-resistant tuberculosis without extensive drug resistance | Infectious (b) |
| HIV/AIDS - Extensively drug-resistant tuberculosis | Infectious (b) |
| HIV/AIDS resulting in other diseases | Infectious (b) |
| Sexually transmitted infections excluding HIV | Infectious (a) |
| Syphilis | Infectious (a) |
| Chlamydial infection | Infectious (a) |
| Gonococcal infection | Infectious (a) |
| Other sexually transmitted infections | Infectious (a) |
| Tuberculosis | Infectious (b) |
| Drug-susceptible tuberculosis | Infectious (b) |
| Multidrug-resistant tuberculosis without extensive drug resistance | Infectious (b) |
| Extensively drug-resistant tuberculosis | Infectious (b) |
| Lower respiratory infections | Infectious (c) |
| Upper respiratory infections | Infectious (c) |
| Otitis media | Infectious (c) |
| Diarrhoeal diseases | Infectious (b) |
| Typhoid fever | Infectious (c) |
| Paratyphoid fever | Infectious (c) |
| Invasive non-typhoidal salmonella | Infectious (c) |
| Other intestinal infectious diseases | Infectious (b) |
| Other intestinal infectious diseases | Infectious (c) |
| Malaria | Neglected tropical diseases |
| Chagas disease | Neglected tropical diseases |
| Leishmaniasis | Neglected tropical diseases |
| Visceral leishmaniasis | Neglected tropical diseases |
| African trypanosomiasis | Neglected tropical diseases |
| Schistosomiasis | Neglected tropical diseases |
| Cysticercosis | Neglected tropical diseases |
| Cystic echinococcosis | Neglected tropical diseases |
| Dengue | Infectious (c) |
| Yellow fever | Neglected tropical diseases |
| Rabies | Neglected tropical diseases |
| Intestinal nematode infections | Neglected tropical diseases |
| Ascariasis | Neglected tropical diseases |
| Ebola virus disease | Infectious (c) |
| Zika virus disease | Neglected tropical diseases |
| Other neglected tropical diseases | Neglected tropical diseases |
| Meningitis | Infectious (c) |
| Pneumococcal meningitis | Infectious (c) |
| H influenzae type B meningitis | Infectious (c) |
| Meningococcal infection | Infectious (c) |
| Other meningitis | Infectious (c) |
| Encephalitis | Infectious (a) |
| Diphtheria | Infectious (a) |
| Whooping cough | Infectious (b) |
| Tetanus | Infectious (b) |
| Measles | Infectious (b) |
| Varicella and herpes zoster | Infectious (c) |
| Acute hepatitis | Infectious (a) |
| Acute hepatitis A | Infectious (a) |
| Acute hepatitis B | Infectious (a) |
| Acute hepatitis C | Infectious (a) |
| Acute hepatitis E | Infectious (a) |
| Other unspecified infectious diseases | Infectious (a) |
| Other unspecified infectious diseases | Infectious (c) |
| Maternal disorders | Maternal and neonatal disorders |
| Maternal haemorrhage | Maternal and neonatal disorders |
| Maternal sepsis and other pregnancy-related infections | Maternal and neonatal disorders |
| Maternal hypertensive disorders | Maternal and neonatal disorders |
| Maternal obstructed labour and uterine rupture | Maternal and neonatal disorders |
| Maternal abortive outcome | Maternal and neonatal disorders |
| Ectopic pregnancy | Maternal and neonatal disorders |
| Indirect maternal deaths | Maternal and neonatal disorders |
| Late maternal deaths | Maternal and neonatal disorders |
| Maternal deaths aggravated by HIV/AIDS | Maternal and neonatal disorders |
| Other maternal disorders | Maternal and neonatal disorders |
| Neonatal disorders | Maternal and neonatal disorders |
| Neonatal preterm birth | Maternal and neonatal disorders |
| Neonatal encephalopathy due to birth asphyxia and trauma | Maternal and neonatal disorders |
| Neonatal sepsis and other neonatal infections | Maternal and neonatal disorders |
| Haemolytic disease and other neonatal jaundice | Maternal and neonatal disorders |
| Other neonatal disorders | Maternal and neonatal disorders |
| Protein-energy malnutrition | Nutritional deficiencies |
| Other nutritional deficiencies | Nutritional deficiencies |
| Lip and oral cavity cancer | Neoplasms (non-blood) |
| Nasopharynx cancer | Neoplasms (non-blood) |
| Other pharynx cancer | Neoplasms (non-blood) |
| Oesophageal cancer | Neoplasms (non-blood) |
| Stomach cancer | Neoplasms (non-blood) |
| Colon and rectum cancer | Neoplasms (non-blood) |
| Liver cancer | Neoplasms (non-blood) |
| Liver cancer due to hepatitis B | Neoplasms (non-blood) |
| Liver cancer due to hepatitis C | Neoplasms (non-blood) |
| Liver cancer due to alcohol use | Neoplasms (non-blood) |
| Liver cancer due to NASH | Neoplasms (non-blood) |
| Liver cancer due to other causes | Neoplasms (non-blood) |
| Gallbladder and biliary tract cancer | Neoplasms (non-blood) |
| Pancreatic cancer | Neoplasms (non-blood) |
| Larynx cancer | Neoplasms (non-blood) |
| Tracheal, bronchus, and lung cancer | Neoplasms (non-blood) |
| Malignant skin melanoma | Neoplasms (non-blood) |
| Non-melanoma skin cancer (squamous-cell carcinoma) | Neoplasms (non-blood) |
| Breast cancer | Neoplasms (non-blood) |
| Cervical cancer | Neoplasms (non-blood) |
| Uterine cancer | Neoplasms (non-blood) |
| Ovarian cancer | Neoplasms (non-blood) |
| Prostate cancer | Neoplasms (non-blood) |
| Testicular cancer | Neoplasms (non-blood) |
| Kidney cancer | Neoplasms (non-blood) |
| Bladder cancer | Neoplasms (non-blood) |
| Brain and nervous system cancer | Neoplasms (non-blood) |
| Thyroid cancer | Neoplasms (non-blood) |
| Mesothelioma | Neoplasms (non-blood) |
| Hodgkin lymphoma | Neoplasms (blood) |
| Non-Hodgkin lymphoma | Neoplasms (blood) |
| Multiple myeloma | Neoplasms (blood) |
| Leukaemia | Neoplasms (blood) |
| Acute lymphoid leukaemia | Neoplasms (blood) |
| Chronic lymphoid leukaemia | Neoplasms (blood) |
| Acute myeloid leukaemia | Neoplasms (blood) |
| Chronic myeloid leukaemia | Neoplasms (blood) |
| Other leukaemia | Neoplasms (blood) |
| Other malignant cancers | Neoplasms (non-blood) |
| Other neoplasms | Neoplasms (non-blood) |
| Myelodysplastic, myeloproliferative, and other haemopoietic neoplasms | Neoplasms (non-blood) |
| Other benign and in situ neoplasms | Neoplasms (non-blood) |
| Rheumatic heart disease | Cardiovascular diseases |
| Ischaemic heart disease | Cardiovascular diseases |
| Stroke | Cardiovascular diseases |
| Ischaemic stroke | Cardiovascular diseases |
| Intracerebral haemorrhage | Cardiovascular diseases |
| Subarachnoid haemorrhage | Cardiovascular diseases |
| Hypertensive heart disease | Cardiovascular diseases |
| Non-rheumatic valvular heart disease | Cardiovascular diseases |
| Non-rheumatic calcific aortic valve disease | Cardiovascular diseases |
| Non-rheumatic degenerative mitral valve disease | Cardiovascular diseases |
| Other non-rheumatic valve diseases | Cardiovascular diseases |
| Cardiomyopathy and myocarditis | Cardiovascular diseases |
| Myocarditis | Cardiovascular diseases |
| Alcoholic cardiomyopathy | Cardiovascular diseases |
| Other cardiomyopathy | Cardiovascular diseases |
| Atrial fibrillation and flutter | Cardiovascular diseases |
| Aortic aneurysm | Cardiovascular diseases |
| Peripheral vascular disease | Cardiovascular diseases |
| Endocarditis | Infectious (c) |
| Other cardiovascular and circulatory diseases | Infectious (a) |
| Other cardiovascular and circulatory diseases | Cardiovascular diseases |
| Chronic obstructive pulmonary disease | Respiratory diseases |
| Pneumoconiosis | Respiratory diseases |
| Silicosis | Respiratory diseases |
| Asbestosis | Respiratory diseases |
| Coal workers pneumoconiosis | Respiratory diseases |
| Other pneumoconiosis | Respiratory diseases |
| Asthma | Respiratory diseases |
| Interstitial lung disease and pulmonary sarcoidosis | Respiratory diseases |
| Other chronic respiratory diseases | Respiratory diseases |
| Cirrhosis and other chronic liver diseases | Digestive diseases |
| Cirrhosis and other chronic liver diseases due to hepatitis B | Digestive diseases |
| Cirrhosis and other chronic liver diseases due to hepatitis C | Digestive diseases |
| Cirrhosis and other chronic liver diseases due to alcohol use | Digestive diseases |
| Cirrhosis due to NASH | Digestive diseases |
| Cirrhosis and other chronic liver diseases due to other causes | Digestive diseases |
| Upper digestive system diseases | Digestive diseases |
| Peptic ulcer disease | Digestive diseases |
| Gastritis and duodenitis | Digestive diseases |
| Appendicitis | Infectious (a) |
| Paralytic ileus and intestinal obstruction | Infectious (a) |
| Inguinal, femoral, and abdominal hernia | Infectious (a) |
| Inflammatory bowel disease | Digestive diseases |
| Vascular intestinal disorders | Digestive diseases |
| Gallbladder and biliary diseases | Digestive diseases |
| Pancreatitis | Digestive diseases |
| Other digestive diseases | Digestive diseases |
| Alzheimer's disease and other dementias | Other non-communicable diseases |
| Parkinson's disease | Other non-communicable diseases |
| Epilepsy | Other non-communicable diseases |
| Multiple sclerosis | Other non-communicable diseases |
| Motor neuron disease | Other non-communicable diseases |
| Other neurological disorders | Other non-communicable diseases |
| Eating disorders | Other non-communicable diseases |
| Anorexia nervosa | Other non-communicable diseases |
| Bulimia nervosa | Other non-communicable diseases |
| Alcohol use disorders | Mental disorders |
| Drug use disorders | Mental disorders |
| Opioid use disorders | Mental disorders |
| Cocaine use disorders | Mental disorders |
| Amphetamine use disorders | Mental disorders |
| Other drug use disorders | Mental disorders |
| Diabetes mellitus | Diabetes and kidney diseases |
| Diabetes mellitus type 1 | Diabetes and kidney diseases |
| Diabetes mellitus type 2 | Diabetes and kidney diseases |
| Chronic kidney disease | Diabetes and kidney diseases |
| Chronic kidney disease due to diabetes mellitus type 1 | Diabetes and kidney diseases |
| Chronic kidney disease due to diabetes mellitus type 2 | Diabetes and kidney diseases |
| Chronic kidney disease due to hypertension | Diabetes and kidney diseases |
| Chronic kidney disease due to glomerulonephritis | Diabetes and kidney diseases |
| Chronic kidney disease due to other and unspecified causes | Diabetes and kidney diseases |
| Acute glomerulonephritis | Diabetes and kidney diseases |
| Skin and subcutaneous diseases | Other non-communicable diseases |
| Bacterial skin diseases | Infectious (c) |
| Cellulitis | Infectious (c) |
| Pyoderma | Infectious (c) |
| Decubitus ulcer | Infectious (c) |
| Other skin and subcutaneous diseases | Other non-communicable diseases |
| Musculoskeletal disorders | Other non-communicable diseases |
| Rheumatoid arthritis | Other non-communicable diseases |
| Other musculoskeletal disorders | Other non-communicable diseases |
| Congenital anomalies | Other non-communicable diseases |
| Neural tube defects | Other non-communicable diseases |
| Congenital heart anomalies | Other non-communicable diseases |
| Orofacial clefts | Other non-communicable diseases |
| Down's syndrome | Other non-communicable diseases |
| Other chromosomal abnormalities | Other non-communicable diseases |
| Congenital musculoskeletal and limb anomalies | Other non-communicable diseases |
| Urogenital congenital anomalies | Other non-communicable diseases |
| Digestive congenital anomalies | Other non-communicable diseases |
| Other congenital anomalies | Other non-communicable diseases |
| Urinary diseases and male infertility | Urinary and gynaecological diseases |
| Urinary tract infections | Infectious (c) |
| Urolithiasis | Infectious (c) |
| Other urinary diseases | Urinary and gynaecological diseases |
| Gynaecological diseases | Urinary and gynaecological diseases |
| Uterine fibroids | Urinary and gynaecological diseases |
| Polycystic ovarian syndrome | Other non-communicable diseases |
| Endometriosis | Urinary and gynaecological diseases |
| Genital prolapse | Urinary and gynaecological diseases |
| Other gynaecological diseases | Urinary and gynaecological diseases |
| Haemoglobinopathies and haemolytic anaemias | Other non-communicable diseases |
| Thalassemias | Other non-communicable diseases |
| Sickle cell disorders | Other non-communicable diseases |
| G6PD deficiency | Other non-communicable diseases |
| Other haemoglobinopathies and haemolytic anaemias | Other non-communicable diseases |
| Endocrine, metabolic, blood, and immune disorders | Other non-communicable diseases |
| Sudden infant death syndrome | Other non-communicable diseases |
| Transport injuries | Injuries (b) |
| Road injuries | Injuries (b) |
| Pedestrian road injuries | Injuries (b) |
| Cyclist road injuries | Injuries (b) |
| Motorcyclist road injuries | Injuries (b) |
| Motor vehicle road injuries | Injuries (b) |
| Other road injuries | Injuries (b) |
| Other transport injuries | Injuries (b) |
| Falls | Injuries (b) |
| Drowning | Injuries (a) |
| Fire, heat, and hot substances | Injuries (b) |
| Poisonings | Injuries (a) |
| Poisoning by carbon monoxide | Injuries (a) |
| Poisoning by other means | Injuries (a) |
| Exposure to mechanical forces | Injuries (b) |
| Unintentional firearm injuries | Injuries (b) |
| Other exposure to mechanical forces | Injuries (b) |
| Adverse effects of medical treatment | Injuries (b) |
| Animal contact | Injuries (b) |
| Venomous animal contact | Injuries (b) |
| Non-venomous animal contact | Injuries (b) |
| Foreign body | Injuries (a) |
| Pulmonary aspiration and foreign body in airway | Injuries (a) |
| Foreign body in other body part | Injuries (a) |
| Environmental heat and cold exposure | Injuries (a) |
| Exposure to forces of nature | Injuries (b) |
| Other unintentional injuries | Injuries (a) |
| Other unintentional injuries | Injuries (b) |
| Self-harm | Injuries (b) |
| Self-harm by firearm | Injuries (b) |
| Self-harm by other specified means | Injuries (a) |
| Self-harm by other specified means | Injuries (b) |
| Interpersonal violence | Injuries (b) |
| Assault by firearm | Injuries (b) |
| Assault by sharp object | Injuries (b) |
| Assault by other means | Injuries (b) |
| Conflict and terrorism | Injuries (b) |
| Executions and police conflict | Injuries (b) |

Abbreviations: COPD=chronic obstructive pulmonary disease. HIV/AIDS=human immunodeficiency virus/acquired immune deficiency syndrome. NASH=non-alcoholic steatohepatitis.

The models used to estimate mortality and incidence used a nested random-effects structure on the underlying cause.

This hierarchical nesting allowed the model to estimate sepsis fractions for mortality and case fatality rates for incidence.

For diseases not represented in the input data, information was borrowed from diseases within the same group. All underlying causes were categorised into 17 groups according to physiological relatedness.

**Supplementary results**

The supplementary implied CFR analysis should be interpreted as a population-level fatality ratio rather than the hospital-based case fatality model used in the 2025 GBD sepsis paper. In this reconstruction, overall implied CFR declined from 63.7% (95% UI 45.8-91.3) in 1990 to 36.6% (22.4-61.1) in 2021 and remained essentially unchanged between 2019 and 2021. In 2021, the implied CFR was highest for sepsis attributed to underlying infection (50.2% [30.9-83.4]), followed by non-communicable diseases (29.1% [17.7-48.8]) and injuries (13.6% [8.3-22.9]). Proxy-rescaled sex-specific estimates suggested a higher 2021 implied CFR in males (43.0% [26.3-71.7]) than in females (30.2% [18.5-50.4]), but these sex-specific values should be interpreted cautiously because they were derived from proportional distributions rather than direct re-estimation of the original GBD hospital model.

**Figure S1. Overall trends in sepsis incidence and mortality in China, 1990-2021.**
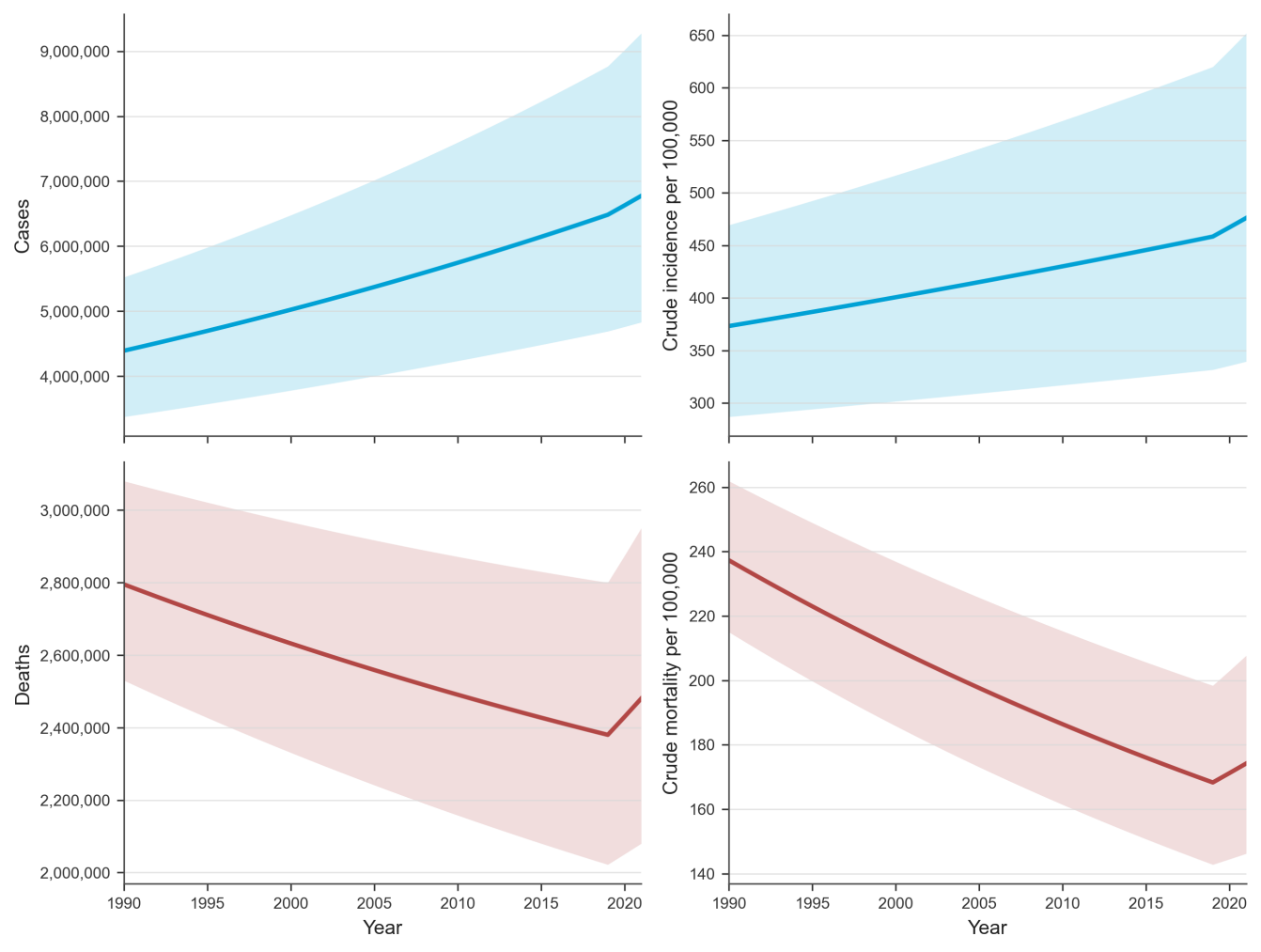


Panel A shows incident sepsis cases; panel B shows crude incidence rates; panel C shows sepsis-related deaths; and panel D shows crude mortality rates.

**Figure S2. Implied case fatality ratio of sepsis in China, 1990-2021, overall and by broad underlying cause category in 2021.**
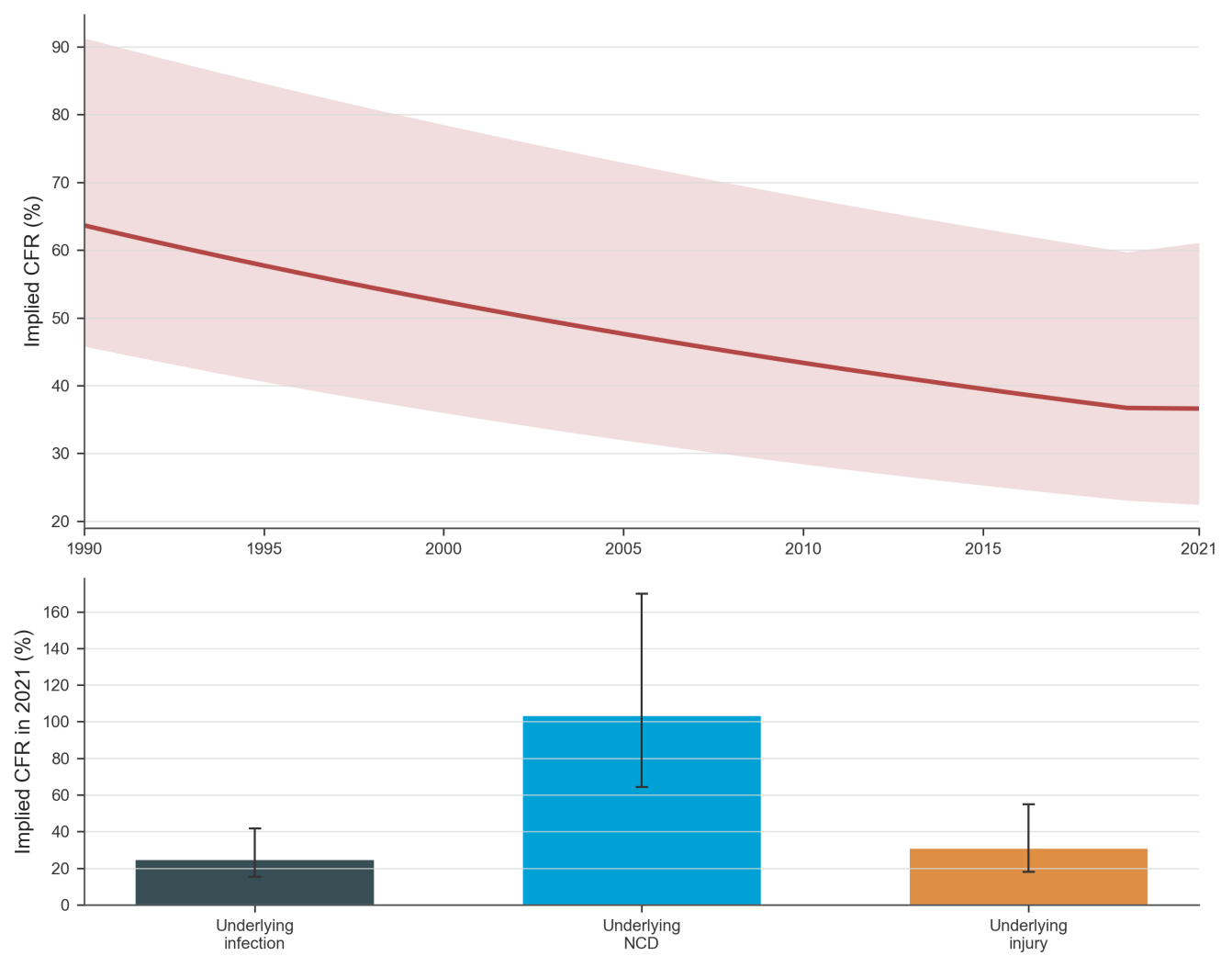


Panel A shows the overall implied case fatality ratio trend; panel B shows the implied case fatality ratio by broad underlying cause category in 2021.

**Figure S3. Age-standardised incidence and mortality of sepsis by broad underlying cause category in China,1990-2021.**
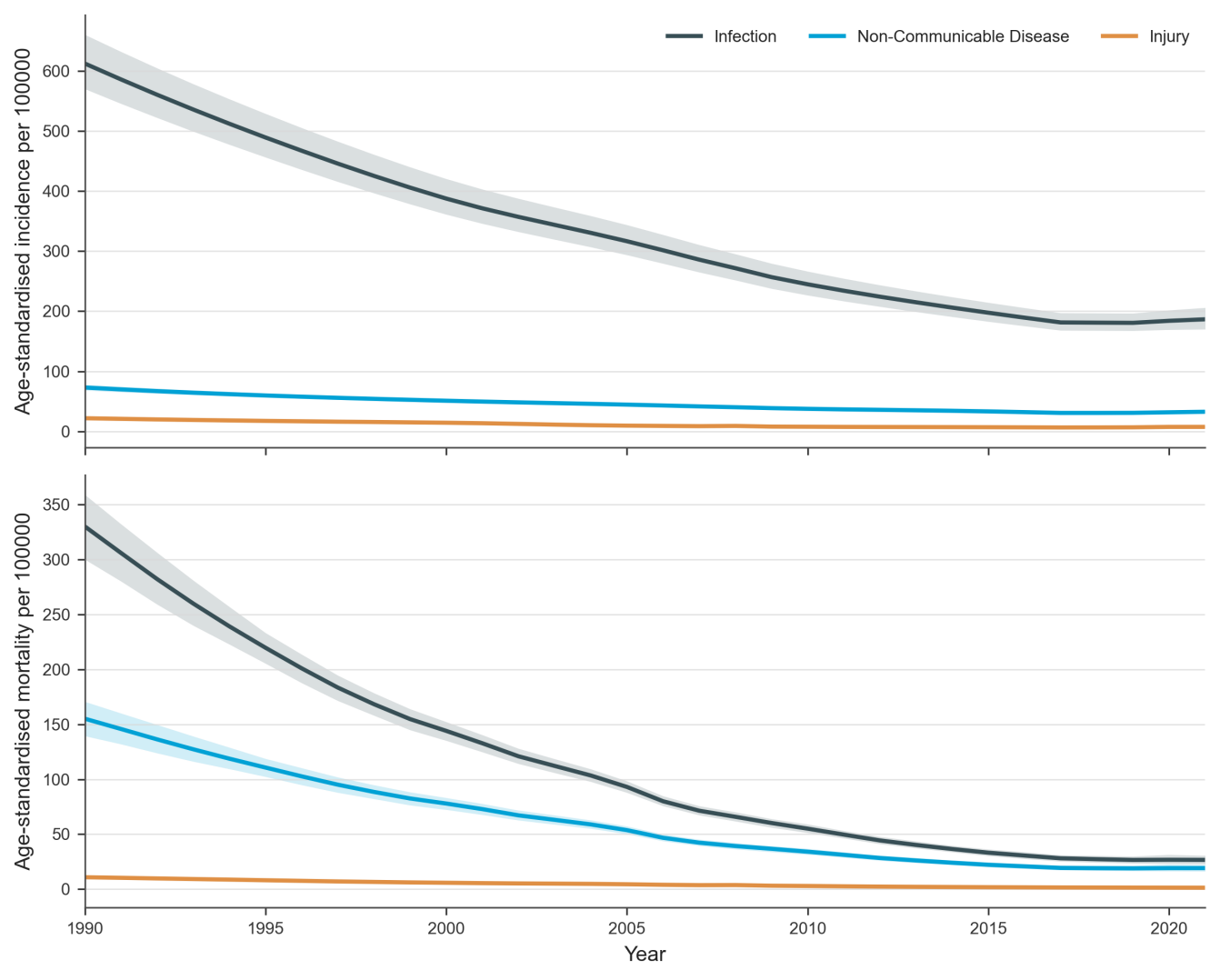


Panel A shows hybrid age-standardised incidence by broad underlying cause category; panel B shows hybrid age-standardised mortality by broad underlying cause category.

**Figure S4. Hybrid age-standardised incidence of sepsis in China, 1990-2021.**
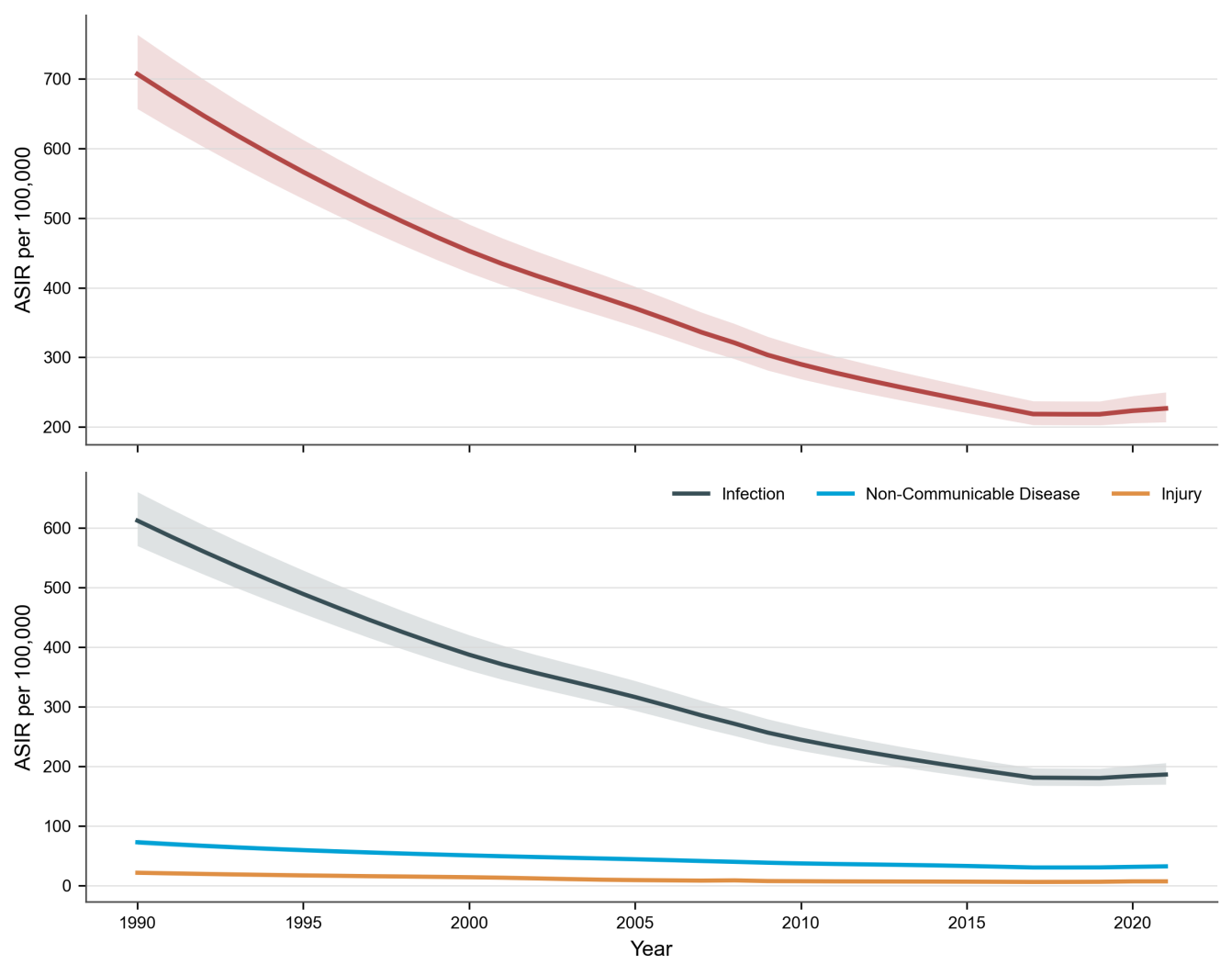


Panel A shows the overall hybrid age-standardised incidence rate; panel B shows hybrid age-standardised incidence rates by broad underlying cause category.

**Figure S5. Hybrid age-standardised mortality related to sepsis in China, 1990-2021.**
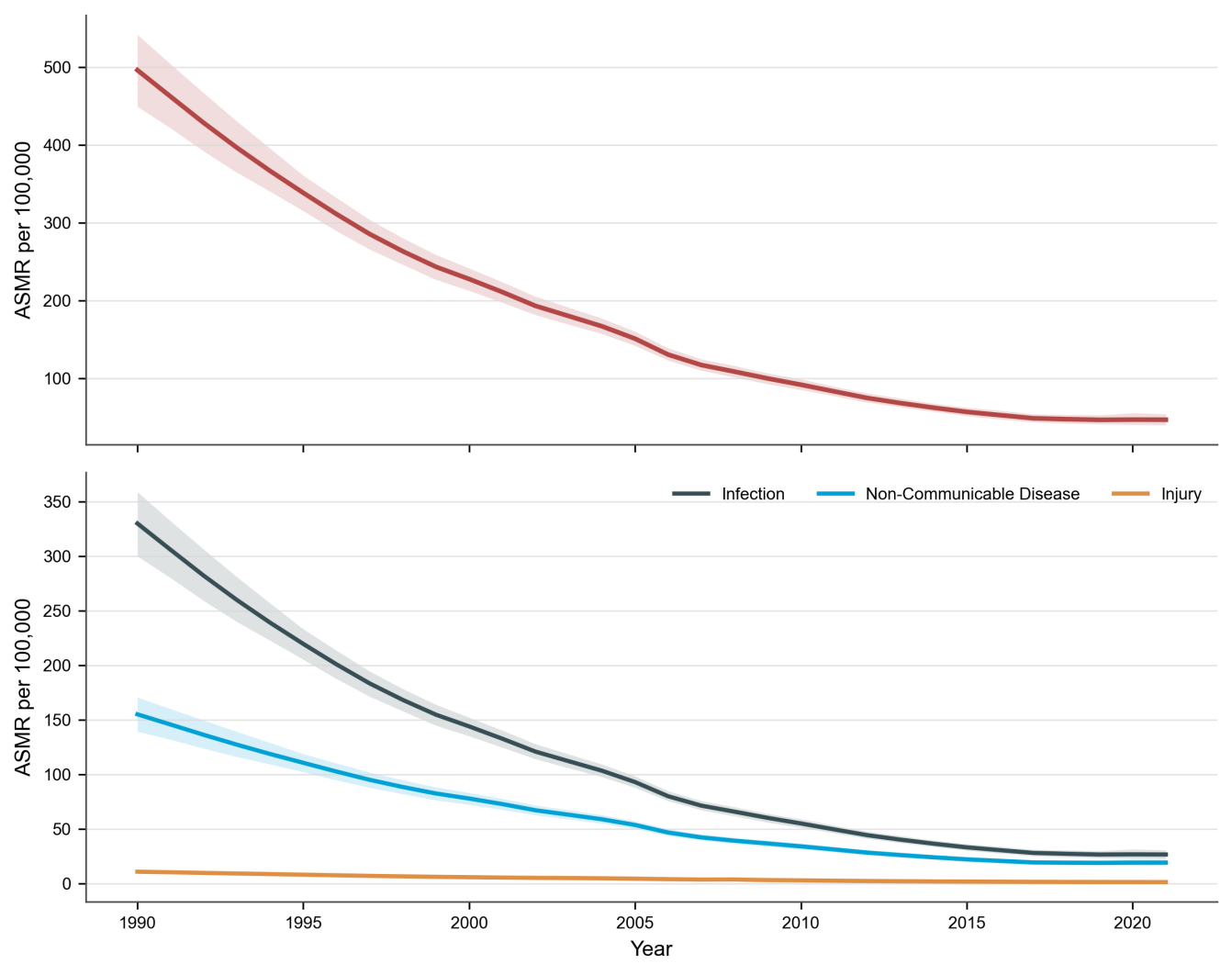


Panel A shows the overall hybrid age-standardised mortality rate; panel B shows hybrid age-standardised mortality rates by broad underlying cause category.

**Figure S6. Trends in sepsis incidence and mortality by broad underlying cause category in China, 1990-2021.**
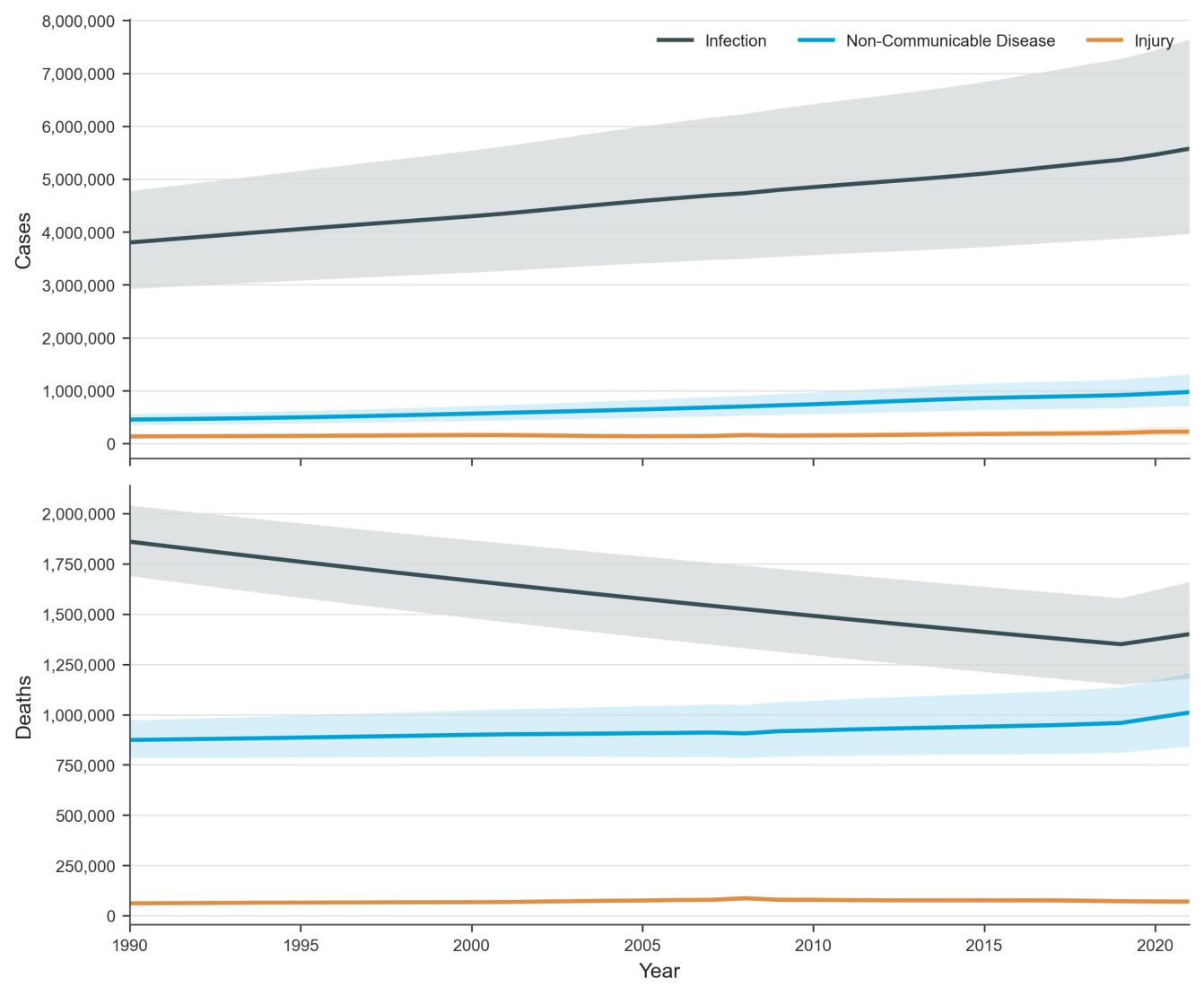
Panel A shows incident sepsis cases by broad underlying cause category; panel B shows sepsis-related deaths by broad underlying cause category.

**Figure S7. Trends in sepsis-related mortality by infectious and non-infectious scope in China, 1990-2021.**
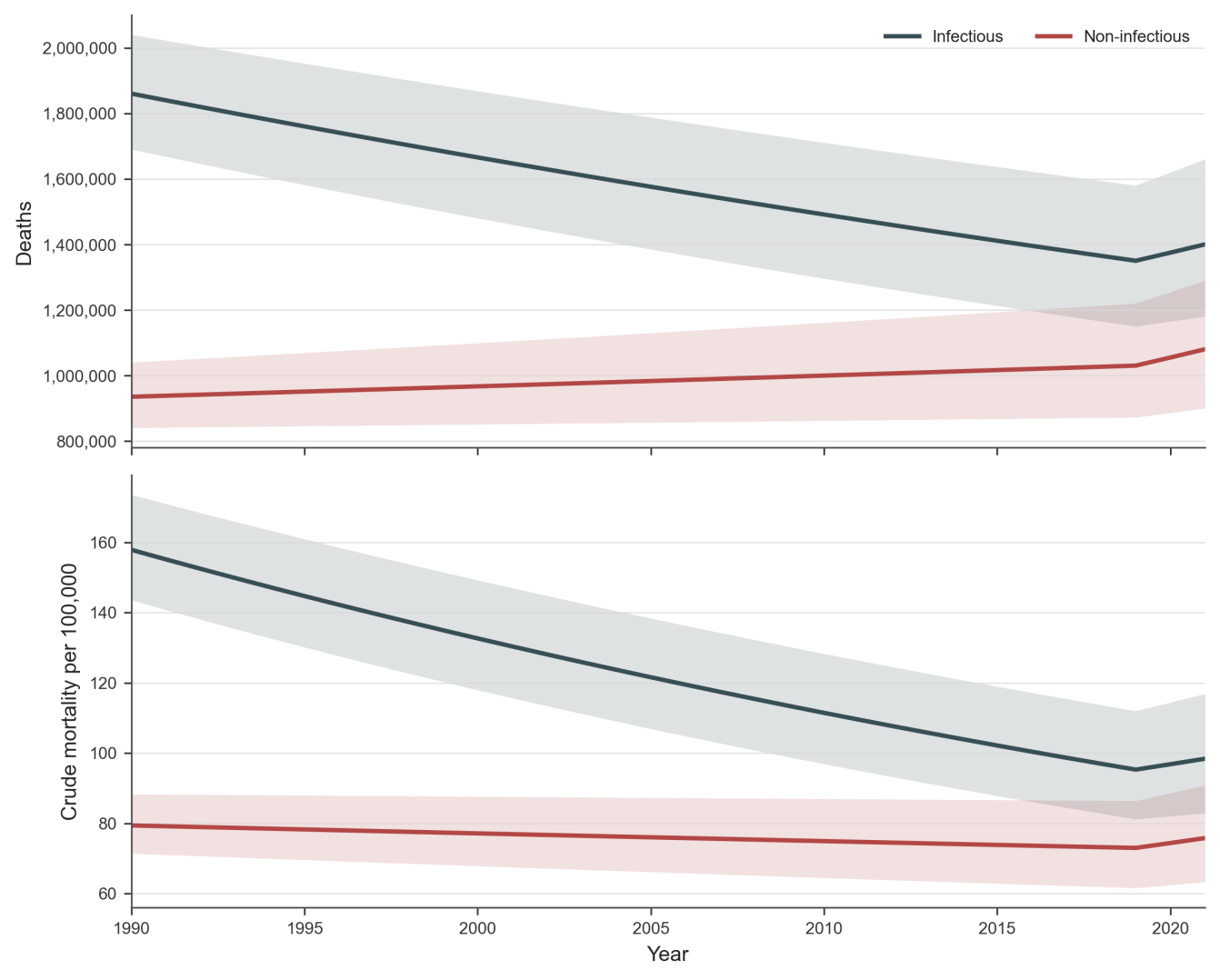


Panel A shows sepsis-related death counts; panel B shows crude mortality rates.
